# Osteotome-Induced Blood Clot and Subsequent Bone Formation with the Use of Collagen Sponge for Integration of Single Dental Implants into the Atrophied Posterior Maxilla: A Retrospective Follow-Up of 36 Implants after 5 to 13 years

**DOI:** 10.1155/2022/6594279

**Published:** 2022-01-05

**Authors:** Stefano Volpe, Michele Di Girolamo, Paolo Pagliani, Sandro Zicari, Lars Sennerby

**Affiliations:** ^1^Private Practice, Rome, Italy; ^2^Department of Periodontology, Tor Vergata University of Rome, Rome, Italy; ^3^Private Practice, Milan and Legnano, Italy; ^4^Department of SARAS, Sapienza University of Rome, Rome, Italy; ^5^Department of Oral & Maxillofacial Surgery, University of Gothenburg, Gothenburg, Sweden; ^6^Private Practice, Clinica Feltre, Feltre, Italy

## Abstract

**Background:**

Atrophy of the posterior maxilla as a consequence of tooth loss and sinus pneumatization is a frequent condition encountered in the clinical practice. Prosthetic rehabilitation with implants in these patients often requires some kind of bone regeneration procedure to increase the bone volume.

**Aim:**

The aim of the present retrospective study is to analyze the survival and success rates of a series of implants placed in the atrophic posterior maxilla with a transcrestal osteotome procedure, without placing a bone grafting material.

**Materials and Methods:**

From 2006 to 2014, 36 dental implants (Neoss Ltd., Harrogate, UK) were inserted in 36 patients with at least 4 mm of bone below the maxillary sinus using transcrestal osteotome sinus floor elevation and placement of collagen sponge below the sinus membrane. ISQ measurements were made after implant placement and at abutment surgery after 4 to 6 months. The vertical bone height (VBH) was evaluated in intraoral radiographs taken prior to surgery and in radiographs from annual check-up appointments 5 to 13 years after implant placement. In addition, marginal bone loss (MBL) was evaluated.

**Results:**

One implant was lost after four years of prosthetic loading. The remaining 35 implants showed no complications and were loaded with single crowns after 4–6 months of healing. All 35 implants showed clinical success after 8.5 ± 2.8 years of prosthetic loading (from 5 to 13 years). The vertical bone height was 5.9 ± 1.4 mm at surgery, 9.7 ± 1.1 mm at second surgery after 4–6 months, and 8.3 ± 1.8 at the follow-up at 8.5 ± 2.8 years (from 5 to 13 years). The implant stability registered was 73.2 ± 6.2 ISQ at the surgery and 75.8 ± 3.9 at the second surgery after 4–6 months.

**Conclusions:**

The present long-term follow-up study showed that the crestal approach for sinus floor bone augmentation without additional bone grafting results in predicable bone formation and high implant survival. The osteotome technique is a valid alternative to the more invasive lateral window technique in single cases with a minimum of 4 mm of VBH below the maxillary sinus.

## 1. Introduction

Different sinus floor augmentation procedures can be used to enable placement and integration of dental implants into the atrophied posterior maxilla. The most common techniques are (i) a lateral approach using an infractured bone window and placement of graft material prior to or in conjunction with implant placement and (ii) a transcrestal technique using osteotomes and simultaneous implant placement [1, 2]. Both techniques have the objective of detaching and elevating the Schneiderian membrane and filling the space with autogenous bone or bone substitutes, with a combination of the two, or with only the blood clot. Summers was the first to describe the transcrestal technique, which was indicated in implant sites with at least 6 mm of bone height between the alveolar ridge and the floor of the maxillary sinus [3]. The technique makes use of a sequential set of osteotomes with increasing diameter. The principle is to prepare an osteotomy up to about one mm below the cortex of the sinus floor. Thereafter, osteotomes of progressively increasing diameter are used to fracture and compact circumferential bone of the osteotomy toward the floor of the sinus. Additional autologous and/or heterologous bone can be added to the implant tunnel at the same time as the sinus floor is lifted with the sinus mucosa where after a dental implant is inserted. The crestal approach is particularly suitable for single tooth replacements, and it is undoubtedly less invasive and creates less postoperative discomfort than the lateral window technique. Conversely, poor visibility increases the risk of making small lacerations if the osteotomes should penetrate excessively into the maxillary sinus [4]. Engelke and Deckver used the crestal approach technique under endoscopic control and concluded that the mucosa can be raised up to 5 mm without risk of lacerations [5]. In a retrospective multicentre study, Rosen et al. evaluated 174 implants in 101 patients inserted with Summer's technique. They reported a survival rate of 96% when the residual bone height was ≥5 mm while it decreased to 85.7% at <4 mm [6].

The use of graft material is reported in most of the works with the aim of acting as a shock absorber during the osteotomy procedure but mainly inducing bone formation around the implant. However, various studies consider the mere blood clot as an effective generator of new bone [7–11]. In 1993, Boyne achieved bone regeneration under the sinus mucosa in an animal model without the use of filler material but by lifting the mucosa and at the same time inserting the implants, with the sole purpose of creating the so-called “curtain effect” [7]. In 1997, Ellegaard et al. placed 38 implants inside the maxillary sinuses of 24 patients, using the lateral window technique. Once the sinus mucosa was lifted, they placed the implants and left the space created to fill with clot. After 5 months of healing, they radiologically found the presence of new bone, and excellent implant stability was found after 27 months of prosthetic loading [8]. The efficacy of using a replaceable lateral bone window, sinus membrane elevation, and simultaneous placement dental implants has been demonstrated both histologically and clinically in many studies [9]. In a study including 17 patients and 25 implants placed with the osteotome technique without filling material, Nedir et al. reported average bone regeneration within the sinuses of 3.1 ± 1.5 mm and no implant failures after 3 years of prosthetic loading [10]. Hence, the use of blood clot alone seems as effective as the use of filling materials with both lateral and crestal approaches. In both surgical techniques, a space is created between the membrane and the floor of the maxillary sinus which is initially filled by the blood clot, and subsequently new bone is formed, following the principle of “GBR” (guided bone regeneration) [11].

An alternative technique to using bone grafts with the osteotome technique is the use of a soft biomaterial, for instance, a collagen sponge, to facilitate the creation of a submembrane space for bone formation [12]. This can be inserted via the crest under the sinus membrane as a space maintaining measure and theoretically to facilitate organization of a blood clot for predictable bone formation. The use of a hydroxyapatite-powdered membrane has been shown to improve the sinus membrane tenting effect and bone formation at implants in the rabbit sinus [13]. The authors also reported that the morphology of the sinus may influence the outcome and suggested that a narrow sinus with bone walls in close proximity is favorable for bone formation [13].

The purpose of this retrospective study is to evaluate bone formation and implant survival 5 to 13 years after transcrestal osteotome-induced sinus floor elevation and simultaneous placement of the implants in 36 consecutive patients.

## 2. Materials and Methods

### 2.1. Patients

The present retrospective patient chart study includes 36 patients with 36 implants (10 men and 26 women, mean age 53 ± 14 years), consecutively treated with a transcrestal sinus floor elevation technique and simultaneous placement of one dental implant to support a single crown in the posterior maxilla by one experienced operator (SV) (Table 1). The study was reviewed and registered by the local ethical committee (Comitato Etico Lazio, Rome, Italy, Prot 739/CE) according to the guidelines for observational retrospective chart studies given by the committee. The STROBE guidelines for reporting of observational studies were followed [14, 15].

The inclusion criteria were as follows:

- Edentulous upper-posterior maxilla with good horizontal and vertical intermaxillary ratios
- Residual bone height of ≥4 mm below the maxillary sinus
- Absence of diseases affecting the maxillary sinus
- Absence of periodontal disease
- Absence of chronic systemic diseases
- Absence of bruxism
- Absence of bisphosphonate intake
- Absence of smoking more than 10 cigarettes a day
- Absence of alcohol abuse

The preoperative evaluation included measurements of the bone height available between the alveolar ridge and the cortex of the sinus floor using orthopanoramic radiography and intraoral X-rays performed (Rinn XCP Instrument Dentsply, York, PA 17404 USA). A parallel technique using an occlusal template was used. Some cases required examination with Cone Bean 3D Imaging System (Morita, Imola, Italy). Before surgery, all patients were informed of the planned surgical technique, possible complications, and benefits and gave their consent to the treatment.

### 2.2. Surgical and Prosthetic Technique

Surgery was performed under local anesthesia (Mepivacaine 2%, Saint-Maur-des-Fossés, France) and with prophylactic antibiotics (amoxicillin + clavulanic acid 1 g, GlaxoSmithKline S.p.A. Milan, Italy). The alveolar bone was exposed via a crestal incision and full thickness flap. The implant bed was prepared with a 2.2 mm twist drill up to the cortex of the maxillary sinus floor, about 1 mm from the previously calculated height of the residual bone in the preoperative X-ray. A depth gauge was inserted into the site, and the working depth was defined by an intraoral X-ray. The preparation of the implant bed then proceeded using the standard sequence of drills for the implant diameter initially chosen, avoiding contact with the sinus mucosa. For a 4 mm diameter implant, the last drill was 3.4 mm and 3.9 mm for a 4.5 mm implant.

The sinus floor fracture was performed by inserting an osteotome (ASA Dental S.p.A., Bozzano, Lucca, Italy) of the same diameter as the last drill used inside the implant socket. A collagen sponge (Condress, Smith & Nephew, Agrate Brianza, Italy) was interposed between the cortex and the tip of the osteotome. A gentle hammering was then initiated until the cortex was perceived to be fractured into the sinus. Some more collagen was added before gently lifting the sinus mucosa with a manual instrument (Maxil, Omnia, Fidenza, Parma, Italy). This phase could be repeated several times until the desired space under the sinus membrane was obtained. The collagen sponges were used both to stabilize the clot inside the chamber created and to prevent the apex of the implant from coming into contact with the mucosa during its insertion, thus reducing the possibility of micro tears. Before inserting the implant, the possible perforation of the membrane was checked with the Valsalva maneuver.

The implant was inserted gently with low rotating speed and sometimes with a manual wrench to avoid damaging the sinus membrane. All implants were placed with the implant platform at the crestal level. Implant stability (ISQ) was assessed using resonance frequency analysis (RFA) measurements (Osstell Mentor, Osstell AB, Goteborg, Sweden) in mesiodistal and buccopalatal directions. Submerged healing with cover screw was adopted. An intraoral X-ray was taken after surgery with customized centering devices (Rinn XCP Instrument Dentsply, York, PA 17404 USA), to obtain superimposable images, standardized over time.

All patients were prescribed antibiotic therapy (Amoxicillin, Sandoz AS, Copenhagen, Denmark, 1 g × 2 for 5 days), antiphlogistics (Ibuprofen, B. Braun Melsungen AG Melsungen, Germany, 400 mg × 2 times for 2 days), and oral antiseptic mouth wash (Corsodyl, GlaxoSmithKline, Brentford, UK) up to 10 days after the removal of the sutures. The sutures were removed after about 10 days.

Each patient underwent periodic check-ups, initially weekly and then monthly to intercept possible complications.

The implants were exposed after 4 to 6 months for healing-abutment connection. A second RFA measurement was made. After a few weeks of healing, a conventional impression was made for fabrication of a metal-ceramic crown, which was attached to the implant within 1.8 ± 1.4 months after abutment connection.

### 2.3. Clinical and Radiographic Follow-Up

**Figure 1:**
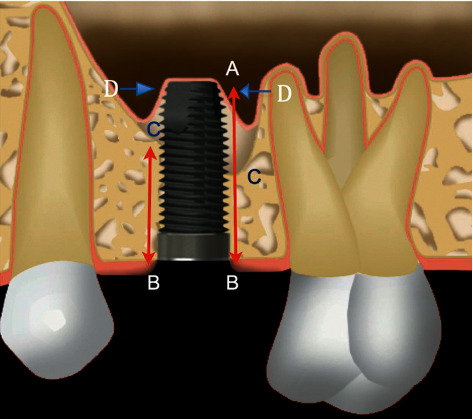
Outline of the parameters measured in the radiographs. *A*  the most apical point of the implant, *B*  the most coronal point of the implant, *C*  the original level of the maxillary floor, *D*  the most apical point of the new maxillary sinus floor. *B*–*C*  height of residual bone below the maxillary sinus before surgery (RBH). *C*-*A*  length of the implant inside the maxillary sinus. *D*  most apical point of new bone in contact with the implant. *D*-*C*  height of bone regeneration within the maxillary sinuses.

After finishing prosthetic treatment, the patients were scheduled for annual clinical check-ups. Follow-up intraoral X-rays from baseline, abutment connection surgery, and the most recent annual check-up were used for calculation of bone regeneration within the maxillary sinus (vertical bone height (VBH)) and marginal bone loss (MBL) by one external specialist. In brief, the VBH was measured from the top of the implant to the highest point of the sinus floor at distal and mesial aspects using a magnifying lens (x4.5, Carl Zeiss, Oberkochen, Germany) and calipers (Figure 1). Mean values from distal and mesial measurements were used to calculate any gain of VBH with time. Likewise, MBL was calculated based on mean marginal bone level measurements from the top of the implant to the first bone contact at mesial and distal aspects.

### 2.4. Statistics

The statistical analysis was conducted using the SPSS software (IBM, Milan, Italy).

Differences between mean values at different time points were tested with the ANOVA test for repeated measurements and with Student's *t*-test for paired data. If they were significant, pairwise comparisons were performed to verify at which time point the parameters differed. A statistically significant difference was considered if *p* ≤ 0.05.

## 3. Results

### 3.1. Clinical Results

A single perforation of Schneider's mucosa was diagnosed by a Valsalva maneuver and was resolved by inserting a shorter implant. No postsurgical complications such as nasal bleeding or sinus infections were experienced.

One implant in the first premolar region was lost after 4 years of prosthetic loading. The remaining 35 implants healed and were loaded throughout the follow-up period without problems, giving a survival rate of 97.2%.

### 3.2. Radiographic Results

**Figure 2:**
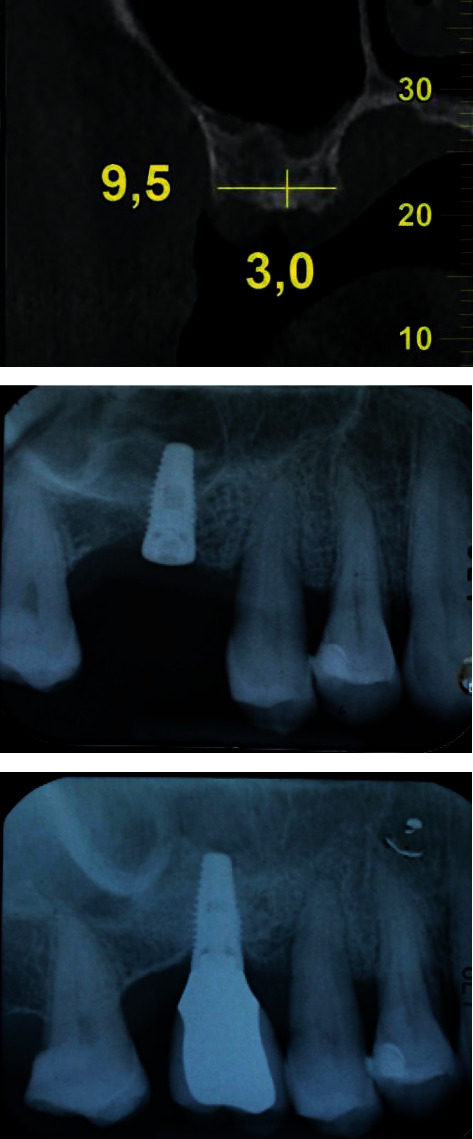
(a) Preoperative CT showing a crestal width of 9.5 mm and a distance of 3 mm from the top of the crest to the lowest point of the maxillary sinus. (b) Periapical radiograph taken immediately after implant insertion. (c) Periapical radiography taken after 9 years of prosthetic loading: note the bone remodeling and the new position of the sinus cortex.

The obtained sinus floor bone augmentation ranged from 2.0 mm to 6.0 mm with a mean of 3.8 ± 1.1 mm (Table 2). The mean VBH was 5.9 ± 1.4 mm at first surgery, 9.7 ± 1.1 mm at second surgery, and 8.3 ± 1.8 mm at follow-up (Table 2). The mean bone gain was found to be statistically significant (*p* < 0.001) both at the second surgery (3.7 ± 1.2 mm) and at the follow-up (2.4 ± 1,4 mm) (Figure 2, Table 2).

The mean marginal bone loss (MBL) at follow-up was 1.0 ± 0.4 mm.

### 3.3. Implant Stability

The stability of the implants increased significantly by 2.6 ± 1.0 ISQ from the first to the second surgery (*p* < 0.001), from 73.2 ± 6.2 to 75.8 ± 3.9 ISQ.

## 4. Discussion

The present retrospective study showed predictable bone formation at the sinus floor following a transcrestal sinus lift procedure and simultaneous implant placement after a mean of 8.5 ± 2.8 years. The radiographic evaluation showed average bone regeneration, obtained with the clot alone without the use of fillers, of 2.4 ± 1.4 mm. Moreover, a mean increase of implant stability of 2.6 ± 1.0 ISQ was observed from placement to abutment connection surgery 4 to 6 month later. Only one implant was lost after 4 years of prosthetic loading, while the remaining 35 implants showed no problems, giving a survival rate of 97.2% after 5 to 13 years. The mean bone loss was 1.0 ± 0.4 mm at follow-up, which is similar to those found in previous studies on the same implant design and followed for at least 5 years [15, 16].

A review based on 19 studies using the osteotome technique demonstrated a survival rate of 95% after 5 years. The most noticeable difference was between implants placed in residual bone height less than 5 mm, which showed survival of 92% compared with 96% for implants placed in bone of 5 mm or more. Furthermore, the authors concluded that the use of filler materials was not relevant to implant survival [17]. This is in line with the results of the present and other studies, where no bone substitutes were used, published after the review study. In a previous study on 29 implants in 20 patients, there were no implant failures, and an average increase in VBH of 2.8 ± 1.1 mm was seen after 11–32 month [18]. Similar results were reported by Fornell et al. who used a CBCT-guided technique in 14 consecutive patients [19]. Preoperative CBCT with a titanium screw post as an indicator at the planned implant position was used to guide a flapless surgical procedure with 21 implants. The implants were followed clinically and with CBCT for 3, 6, and 12 months postoperatively. The implants penetrated on average 4.4 ± 2.1 mm into the sinus cavity, the change of VBH was 3 ± 2.1 mm, and none of the implants failed after one year.

The biological basis for new bone formation process below the sinus mucosa follows the principles of bone formation in general and, for instance, the healing events seen in postextraction sockets [20]. The blood clot induces growth, proliferation, and differentiation of various types of cells by stimulating angiogenesis and formation of new bone. The curtain effect of the implant and the mucosa triggers a process that resembles the principles of the GBR technique, where a space is secluded with a barrier to allow for bone formation. The periosteum is an important source of cells for induction of new bone formation following a trauma like implant placement. However, in the maxillary sinus, the sinus membrane has also been reported to play an important role in bone regeneration. Srouji et al. demonstrated *in vivo* and *in vitro* that Schneider's mucosa is a potential source of multipotent mesenchymal stem cells that are crucial for the healing process. [21] In 2006, Palma et al. were the first to histologically demonstrate bone regeneration below the sinus mucosa supported by implants without filler materials. They concluded that the membrane plays a fundamental role in regeneration both for its intrinsic properties and for the barrier function to protect the clot [22]. However, in a later study using the same animal model from the same group, bone formation was seen to start from the bottom of the sinus floor, and no bone formation could be seen in conjunction with the sinus membrane after 10 days of healing [23]. This is in line with Scala et al. who showed that the sinus membrane does not participate in the early regenerative process and that the input starts from the bone of the sinus floor and from the bone micro fragments carried inside it during insertion [24].

One question of interest is the potential risks with leaving exposed implant apices in the maxillary sinus cavity since bone is normally not completely covering this part of the implants with or without the use of grafting materials. For instance, Hatano et al. observed a decrease of the grafted bone/bovine bone granule mix from above and below the apices of the implants during the first three years after the surgery [25]. Volpe et al. used the same technique as in the present study and noted that, immediately after surgery, the mean membrane elevation was on average 4.5 mm, while at the follow-up after 5–24 months of loading, the bone regeneration was on average 3.5 mm [26]. Similar results were found in the present patient group, and this can be the consequence of the pressure exerted by the sinus membrane during breathing on the blood clot and on the interposed collagen. It is possible that the movements of the membrane prevent stabilization of the clot and consequently the formation of new bone above the implant tips. However, a follow-up study on lateral sinus floor augmentation cases using CBCT found that the implant tips often protrude through the grafted area but are covered with a healthy sinus membrane [27]. Other studies have reported similar results and that the apical implant part was covered with a seemingly healthy sinus membrane [28–31]. Moreover, follow-up studies of zygomatic implants penetrating the maxillary sinus have reported few problems related to the exposed implant surface [32].

A parallel technique with an occlusal template, as originally described by Gómez-Roman et al. and commonly used in clinical research, was used to standardize the intraoral radiographs [33]. Nevertheless, the problem of taking a perfectly standardized series of radiographs over time still exists as the occlusal anatomy may change over time and the position of the X-ray cone may vary despite the template. Moreover, it has been shown that linear measurements of bone levels at implants in intraoral radiographs are less accurate than direct measurements during surgery [34] or in histological sections [35]. The deviation from true marginal bone levels, as established by direct clinical or histologic measurements, and linear measurements in intraoral radiographs of the same implants is around 0.4–0.5 mm according to comparative studies [34, 35]. However, for obvious reasons, it was not possible to use any of the techniques in the present retrospective chart study. Since the implant part protruding into the maxillary sinus is not masked by surrounding preexisting bone, it can be argued that measurements in this situation may be more accurate than when measuring marginal bone levels. Moreover, the purpose of the present study was to show that bone formation occurs when using a collagen sponge in conjunction with the osteotome technique rather than establishing the exact amount of bone. In this sense, the measurements from observational study of one group of patients were used as a descriptive parameter and not to compare different techniques.

In conclusion, within the limitations of the present study, our findings confirm that the osteotome technique used results in predictable bone formation in the sinus and implant integration without the use of any bone fillers. The technique is a valid alternative to the more traumatic lateral approach technique, at least for single tooth replacements with more than 4 mm of bone below the sinus.

## Figures and Tables

**Table 1:** Patients and implants.

		Number	Percent (%)
Gender	M	10	27.8
	F	26	72.2

Neoss implant type	Straight	17	47.2
	Tapered	19	52.8

Implant surface	Bimodal	11	30.6
	ProActive	25	69.4

Implant length (mm)	9.0	25	69.4
	11.0	10	27.8
	13.0	1	2.8

Implant diameter (mm)	4.0	23	63.9
	4.5	9	25.0
	5.0	4	11.1

**Table 2:** Results from measurements of intrasinus bone formation.

	Vertical bone height (mm ± SD)	Bone gain from implant surgery (mm ± SD)	Statistics (*P* value)
Implant surgery	5.9 ± 1.4		
Second implant surgery	9.7 ± 1,1	3.8 ± 1.1	0.001
Follow-up	8.3 ± 1.8	2.4 ± 1.4	0.001

## Data Availability

Data can be made available upon request.
